# Unraveling the core symptoms of mental health in senior grade three students- a network analysis

**DOI:** 10.3389/fpsyt.2024.1364334

**Published:** 2024-04-22

**Authors:** Guoxiang Fang, Ying Wang, Huiling Yuan, Ne Yan, Shaomin Zhi

**Affiliations:** ^1^Department of Emergency, Third Hospital of Xi’an, The Affiliated Hospital of Northwest University, Xi’an, Shaanxi, China; ^2^Department of Psychiatry, Xi’an International Medical Center Hospital, The Affiliated Hospital of Northwest University, Xi’an, Shaanxi, China; ^3^Department of Psychology, Xi’an Physical Education University, Xi’an, Shaanxi, China

**Keywords:** mental health, network analysis, core symptoms, senior grade three students, GGM

## Abstract

**Background:**

Adolescence is not only an important transitional period of many developmental challenges, but also a high risk period for mental health problems. Psychotherapy is recommended for mental health problems in adolescents, but its effectiveness is not always satisfactory. One possible contributing factor may be the lack of clarity surrounding core symptoms.

**Methods:**

In this study, we investigated the mental health status of senior grade three students, a group of adolescents facing college entrance exams, by the Middle School Student Mental Health Test (MHT) and analyzed the core symptoms by network analysis. This study was conducted through an online survey platform (www.xiaodongai.com) from 15 February 2023 to 28 March 2024. The subjects scanned a QR code with their mobile phone to receive the questionnaire.

**Results:**

The mean age of these 625 students were 18.11 ± 2.90 years. There are 238 male participants and 387 female participants. 107 individuals scored above 56 (107/461, 23.2%), with individual scale scores over 8 up to over 60% of participating students. Notably, the top three prominent symptoms were “academic anxiety”, “allergic tendency” and “somatic symptoms”. However, upon conducting network analysis, it became evident that three strongest edges in this network were “somatic symptoms” and “impulsive tendency”, “academic anxiety” and “social anxiety” as well as “social anxiety” and “Loneliness tendency”. “somatic symptoms”, “social anxiety” and “self-blame tendency” exerted the highest expected influence. This suggests that, statistically speaking, these three symptoms exhibited the strongest interconnections within the network.

**Limitation:**

Cross-sectional analysis; Bias in self-reported variables.

**Conclusion:**

These findings can deepen the knowledge of mental health among senior grade three students and provide some implications (i.e., targeting symptoms having highest expected influence) for clinical prevention and intervention to address the mental health needs of this particular group.

## 1 Introduction

Adolescence is an important period of rapid physical and mental development, accompanied by numerous developmental challenges (1). As children get older, they engage more deeply in complex social interactions and typically experience a decrease in study motivation, faced with more and more important academic demand (2). prolonged exposure to negative social interactions and academic problems leads to high risk for the onset of mental health problems (3, 4). Evidence shows that most mental health problems arise in childhood and adolescence (5). According to the meta-analysis of the worldwide prevalence of mental disorders in children and adolescents, approximately 11.3-15.9% experience mental disorders (6). So, identifying the mental health needs of young people is crucial to improving prevention. Previous study has shown that mental health issues among high school students are generally severe, provided they are distinguished from junior high school students. During the COVID-19 lockdown in China, sad Mood was the most central symptom among junior high school students, senior high school students. Unlike junior high school students, senior high school students tend to exhibit more suicidal ideation (7). Senior high school students represent a special psychological group. They are in a critical period of physical and mental development, coupled with the stress of the highly competitive College Entrance Examination, one of the most rigorous assessments in China (8). Thus, Senior high school students are uniquely vulnerable to anxiety due to the combination of academic pressure, social challenges, and changing identities. These problems will be more pronounced especially in the senior grade three students. Poor mental health among adolescents not only lead to personal suffering and family burden, but also have potential negative impacts on social development (9). Hence, there is an urgent imperative for surveys to pinpoint problems and direct mental health services appropriately.

With the growing understanding of the Bio-psycho-social model of medicine, we have begun to focus on the impact of psychological, socio-environmental and natural environmental factors on human health. Psychotherapy has been proven to be an effective theoretical technique, widely used in child and adolescent mental health and mental health promotion practices (10, 11). It is recommended for mild depressive disorders in children and adolescents (12). but there is no unified standard for the types and parameters of psychotherapy (13). Clinical opinions differ on the efficacy of psychotherapy and the question of for whom is effective have been the focus of Psychiatrists and psychologists in the past five years (14). Grasping the focus of psychological problems becomes an important influence on the effectiveness of psychotherapy.

To understand the characteristics of adolescent mental health and the need for psychotherapy and, based on this, to propose a model of mental health services that better meets the needs of adolescence. In recent years, network analyses have provided a way to understand the mental health characteristics of adolescents and have been widely used in the field of psychopathology (10, 15). This perspective asserts that mental disorders arise from direct interactions among symptoms (16). This methodology treats symptoms as nodes in a network model and gives the centrality and predictability of the corresponding nodes to understand the interactions between symptoms (17, 18), revealing the core symptoms of psychiatric disorders and providing potential targets for clinical interventions. In addition, network modeling provides a new perspective for understanding co-morbidity (19).

To deepen the knowledge of mental health among senior grade three students and provide some implications (i.e., targeting symptoms having highest expected influence) for clinical prevention and intervention to address the mental health needs of this particular group, we conducted this study to (1) investigate the network structure of mental health symptoms for senior grade 3 students by Middle School Student Mental Health Test (MHT). (2) Clarifying the top central symptoms; and (3) presenting the strongest symptom-to-symptom interaction in network.

## 2 Methods

### 2.1 Participants

Our study was conducted through an online survey platform (www.xiaodongai.com) from 15 February 2023 to 28 March 2024. A total of 680 Chinese senior grade three students from Xi’an city participated in the investigation. The subjects scanned a QR code with their mobile phone to receive the questionnaire. Data collection was done through on-site code scanning, and participants were thanked by the investigator after confirming completion on-site The initial section of the survey primarily comprised of anonymous and informed consent. After reading the informed consent, participants could click “I agree” to complete the following items. The study protocol was approved by the Chinese Clinical Trial Ethics Committee (NO. 2021009; 29 March 2021) and was registered with the Chinese Clinical Trial Registry (NO. ChiCTR2100046396).

### 2.2 The middle school student mental health test

The Middle School Student Mental Health Diagnostic Test (MHT) is a tool designed to assess students’ mental health status. They were majored in by Professor Zhou Bucheng (1991) from the Department of Psychology at East China Normal University. The MHT comprises of 100 questions, categorized into 8 subscales along with a lie detection scale, using a 0-1 scoring system. The eight content scales are as follows: academic anxiety, Anxiety about People, Loneliness Tendency, Self-blame Tendency, Allergy Tendency, somatic symptoms, Horror Tendency and Impulsive Tendency. A score of 8 or above indicates a clear tendency towards psychological problems. No tendency towards psychological problems with a score of 3 or less. The scale categorizes the level of mental health into three grades: a score of 1-55 is considered normal; 56-64 is considered poor mental health and a moderate tendency to psychological problems; and a score of more than 65 is considered a severe tendency to psychological problems. The half reliability of this test tool ranges from 0.84 to 0.88, and the retest reliability is between 0.667 and 0.86, with a validity greater than 0.71.

For the data collected, incomplete data are first eliminated. Secondly, If the score of the lie detection subscale falls between 7 and 10, the test is deemed unreliable, this part of the data should also be excluded.

### 2.3 Network analysis

In network analysis, nodes represent the symptoms and edges represent the relationship between two nodes. The association between two nodes is calculated by partial correlation analysis, keeping the interference of other nodes constant.

#### 2.3.1 Regularized partial correlation network

Statistical analysis and visualization of regularized partial correlation networks are conducted using the software R. The network was estimated using a Graphical Gaussian Model (GGM) (20). The Gaussian Graphical Model (GGM) is used to fit the data and compute the network. GGM is an undirected network where edges represent partial correlations between nodes when all other nodes in the network are controlled for. Estimating GGM requires a large number of parameters, so the graph LASSO (Least Absolute Shrinkage and Selection Operator) algorithm is used to regularize GGM (21, 22). In this regularization process, all edges are reduced, and some edges with low correlation are set to zero, resulting in a more stable, sparse, and easily interpretable network. This algorithm positions symptoms with stronger connections closer to the center of the network, while symptoms with weaker connections are placed closer to the periphery. Thicker edges represent stronger correlations between two symptoms, while thinner edges represent weaker correlations between two symptoms.

#### 2.3.2 Centrality and predictability analysis

Degree centrality, closeness centrality, betweenness centrality, and predictability are calculated to quantify the importance and controllability of each symptom in the regularized partial correlation network. Higher values of centrality indices represent greater centrality. Degree centrality may be the most important and meaningful centrality measure, as nodes with high degree centrality are more likely to influence the entire node group when activated. Additionally, predictability can indicate the controllability of each node. These measures are calculated using the R package qgraph (18).

#### 2.3.3 Network accuracy and stability

R-package bootnet was employed to assess the accuracy and stability of network (20). The accuracy of edge weights was estimated by constructing a 95% confidence interval (CI) around each edge by using bootstrap approach with 2000 samples and computing bootstrapped difference tests for edge weights. The narrower the confidence interval, the more accurate the estimation of edge weights, and the more accurate the estimation of centrality indicators. Moreover, the stability of node expected influences and strength was examined by calculating the correlation stability (CS) coefficient. The value of the CS coefficient should not be less than 0.25 and preferably should be more than 0.5 (20).

## 3 Results

### 3.1 The mental health status for senior grade three students

In this study, we obtained 680 records of data, of which 23 were excluded due to incompleteness, 32 were excluded due to validity values of more than 7 points, and finally 625 valid data were obtained The effective rate of this study was 91.91%. The study comprised 238 male participants and 387 female participants. **Table 1** provides a detailed overview of the prevalence and the detection of various mental health problems for senior grade three students. The total score of 625 students on MHT test for senior grade three students was 46.55 ± 19.08; Among the 625 students, 212 (212/625, 33.9%) scored 56 and 118 scored above 65 (118/625, 18.9%), indicating potential mental health issues. The top three prominent symptoms were “academic anxiety”, “allergic tendency” and “somatic symptoms” (**Table 1**) and the percentage of students scoring more than 8 points accounted for 64.8%, 41.0%, and 36.8% of the total number of participants, respectively. In the study, the mean scores, standard deviations, skewness, kurtosis, expected influence (z-scores) and predictability for each symptom of all MHT items were examined (**Table 1**).

**Table 1:** Descriptive statistics for the network variables the mean scores, standard deviations, expected influence (z-scores) and predictability for each symptom.

Items	Node	M	SD	EI	Skew	Kurt	Pre	No. (%)
**MHT1**	Learning Anxiety	8.87	3.60	-0.70	-0.36	-0.75	0.71	405(64.8%)
**MHT2**	Social Anxiety	5.15	2.73	1.10	-0.01	-0.93	0.64	147(23.5%)
**MHT3**	Loneliness Tendency	3.63	2.95	-1.14	0.50	-0.90	0.75	93 (14.9%)
**MHT4**	Self-blame Tendency	5.37	2.97	1.01	-0.17	-1.09	0.64	184(29.4%)
**MHT5**	Allergy Tendency	6.34	2.44	-0.24	-0.78	0.13	0.70	230(36.8%)
**MHT6**	Physical Symptoms	6.68	3.57	1.30	0.13	-0.74	0.63	256(41.0%)
**MHT7**	Horror Tendency	3.34	2.74	-1.08	0.51	-0.73	0.76	53 (8.5%)
**MHT8**	Impulsive Tendency	3.96	2.89	-0.25	0.24	-1.11	0.71	83 (13.3%)

M, mean; SD, standard deviation; EI, expected influence (z-scores); Skew, skewness; Kurt, kurtosis; Pre, predictability; No. (%), the number and percentage of participants scoring more than eight (≥8 scores) on each symptom.

### 3.2 Network structure of MHT symptoms

**Figure 1:**
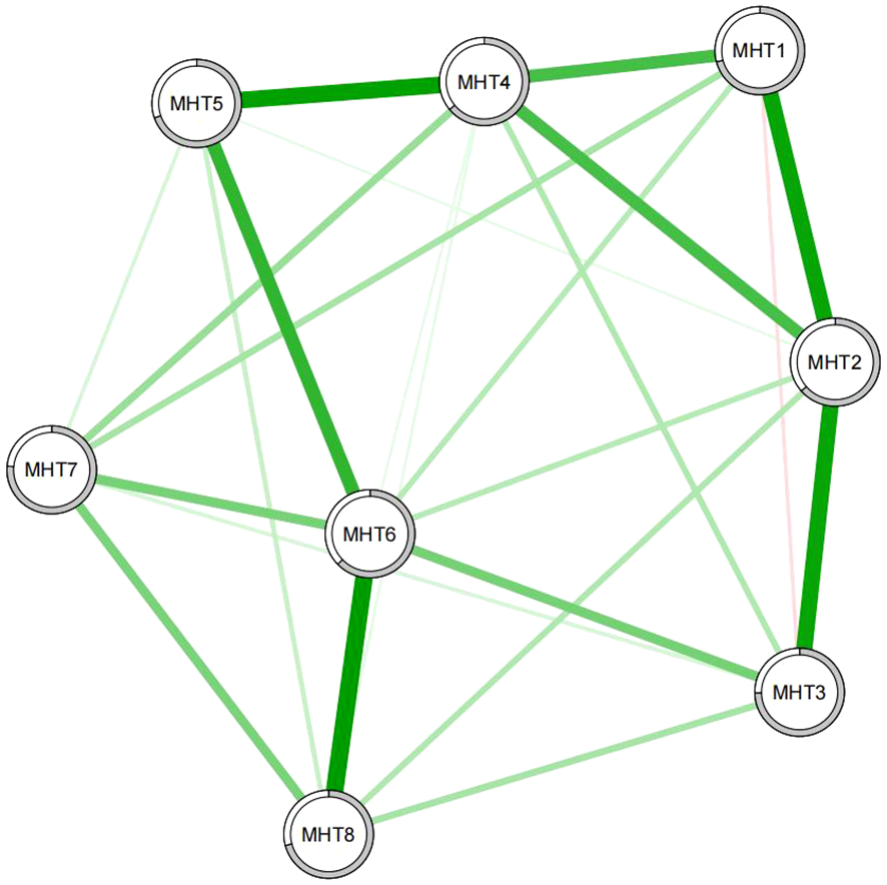
Network structure of MHT symptoms among senior grade three students. Green edges represent positive correlations, red edges represent negative correlations. The thickness of the edge reflects the magnitude of the correlation. The circles around nodes depict its predictability. MHT1, Academic anxiety MHT2, Social anxiety; MHT3, Loneliness tendency; MHT4, Self-blame tendency; MHT5, Allergic tendency; MHT6, somatic symptoms; MHT7, Phobic tendency; MHT8, Impulsive tendency.

**Figure 2:**
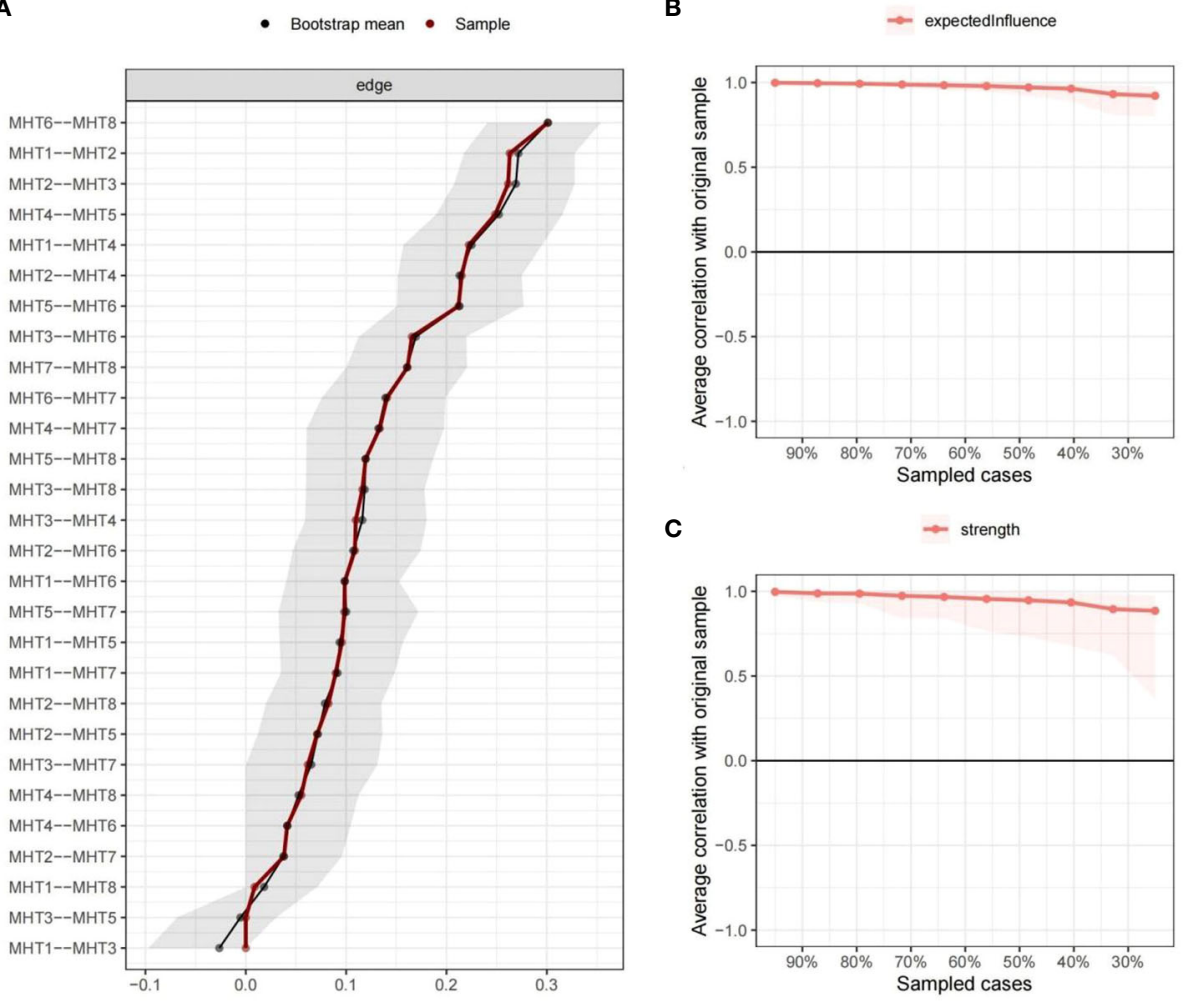
Network Accuracy and Stability. The accuracy of edge weights **(A)** and Stability coefficients associated with the expected influence **(B)** and strength **(C)** in this network were calculated.

The **Figure 1** illustrates the network of MHT symptoms. All 28 edges are positive except the edge between MHT1 “academic anxiety” and MHT3 “Loneliness tendency” (weight = -0.05). Notably, there exist significant regularized bias correlations between the symptoms MHT6 “somatic symptoms” and MHT8 “impulsive tendency” (weight = 0.31), MHT1 “academic anxiety” and MHT2 “social anxiety” (weight = 0.29) as well as MH2 “social anxiety” and MHT3 “Loneliness tendency” (weight = 0.29), Bootstrapped 95% confidence interval indicating the accuracy of edge weights was relatively reliable and accurate (**Figure 2A**).

Node predictability is visualized as circles around the nodes in **Figure 1**. The values of node predictability range from 63% to 75% with an average value of 69%. This indicates that, on average, 69% of the node variance in the network can be explained by its neighboring nodes.

**Figure 3:**
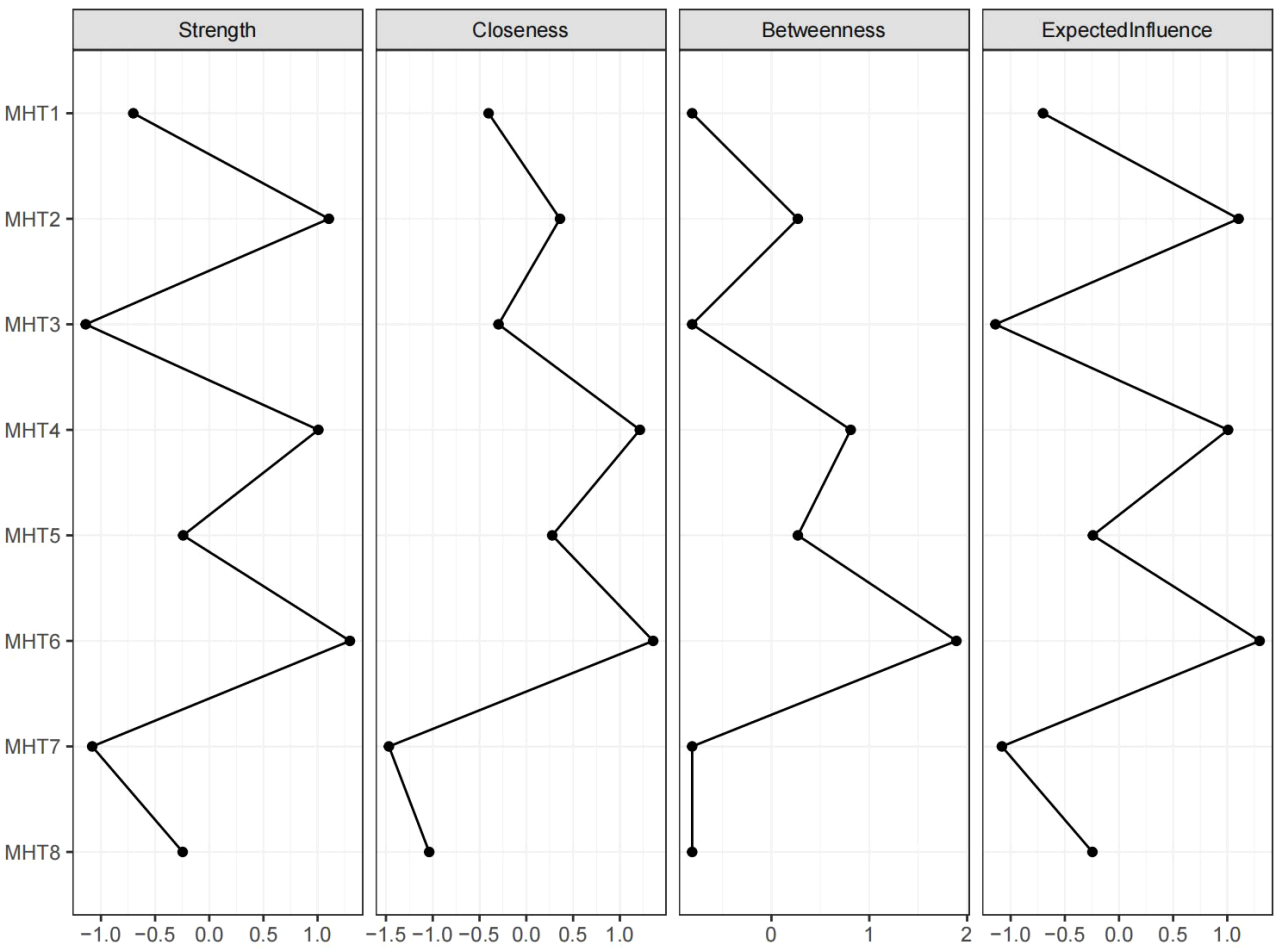
The Centrality and Predictability in the network. Four centrality metrics including strength, betweenness, closeness, and expected influence of each symptom in the network.

**Figure 3** displays the expected influence scores (Z-scores) of the symptoms within the mental health network. “somatic symptoms” have the highest node strength in the symptom network among students, followed by “social anxiety”, and “self-blame tendency”, indicating that these three symptoms are the most associated symptoms in the present network from the perspective of statistics. In **Figures 2B, C**, the stability coefficients for the expected influence and strength are determined to be 0.75 and 0.67 respectively, indicating a stable and reliable estimation of the expected influence of nodes in this study.

## 4 Discussion

In our study, 23.2% of students reported poor mental health and more than 60% of participants suffer from academic anxiety. upon conducting network analysis, three strongest edges in this network were “somatic symptoms” and “impulsive tendency”, “academic anxiety” and “social anxiety” as well as “social anxiety” and “Loneliness tendency” and “somatic symptoms”, “self-blame tendency”, and “social anxiety” exerted the highest expected influence. These findings develop a novel framework for understanding the mental status of these special population and provide some implications for clinical prevention and intervention to address the mental health needs of this particular group.

The World Health Organization defines mental health as “a state of well-being in which individuals are able to appreciate their abilities, cope with the normal stresses of life, work productively and contribute to their communities” (23). Evidence suggests that students in China’s education system are under tremendous academic and competitive pressure (24). The educational stress hypothesis suggests that the expansion of education and the shift to a knowledge-based economy have heightened the significance of academic performance in adolescents’ life prospects. This, in turn, has led to increased academic stress and subsequently, mental health challenges. Previous studies show that 10-20% of children and adolescents suffer from mental health problems (25). Consistent with previous studies, our study revealed that out of 625 students, 107 individuals scored above 56 (107/461, 23.2%), indicating potential mental health problems. More than 60% of the students exhibited symptoms of “academic anxiety”, 41% displayed “somatic symptoms” and 36.8% of them have “allergic tendency”. Notably, academic anxiety has become a major concern for students and parents, as well as a pressing issue for psychotherapists to address in the treatment process (24, 26).

However, treatment is not as effective as it could be, and there are certainly many influencing factors, one of which may be that academic anxiety is something that, although a prominent symptom faced by adolescents, but is not necessarily the core symptom. This is supported by the findings of our study. Through network analysis, we examined the centrality of symptoms. As we know, the expected influence centrality of node plays an important role in finding symptoms that activate or maintain psychopathological networks as well as providing potential targets for intervention (27). The results showed that “somatic symptoms” has the highest centrality, which indicates this symptom play the most important role in activating and maintaining the psychopathology network of MHT in senior grade three students.

Therefore, interventions targeting “somatic symptoms” might generally alleviate MHT symptoms in senior grade three students. This centrality result is consistent with previous studies which investigated symptoms network of Acute stress reaction (ASR) among military college students who were about to participate in an important physical fitness test (28). Stress predicts somatic symptoms in students under academic stress (29). The prevalence of somatic symptoms ranged from 5.7 to 80.1%, and somatic symptoms were overwhelmingly found to be significantly correlated with mental ill-health (30). A previous study has shown that physical reactions may be one of the most symptoms of learning stress during the COVID-19 pandemic (31).

Symptoms “social anxiety” and “self-blame tendency” are two other high centrality symptoms in the present network. Social anxiety is a common type of anxiety among adolescents, characterized by an unreasonable fear of negative assessments in social situations (32). Students exposed to intense pressure (33), which is often linked elevated anxiety levels (34–36). In sociocultural situations with closely knit social networks, worries about being rejected and losing vital social resources may be exacerbated (37). In this case, Chinese adolescents may be more afraid of negative evaluations from peers or teachers. As a result, they are unable to perceive themselves accurately, which can lead to low self-esteem and even feelings of inferiority. Individuals with high levels of low self-esteem often avoid social interactions due to a fear of rejection and tend to adopt an avoidant approach, which can exacerbate social anxiety (38, 39). Chinese adolescents tend to avoid society if they are concerned about negative perceptions of themselves. Feelings related to self-esteem, academic ability, appearance, and physical prowess may directly or indirectly influence social anxiety through fear of negative evaluation (3). Studies showed that self-blame was one of the most frequently used unhealthy coping strategies among physician assistant students (40) and medical students (41).

In this study, the strongest correlation was between “somatic symptoms” and “impulsive tendency”. The prevalence of somatic symptoms was 65.10% among the adolescents experiencing school-related stressors (42).

There exists a positive association between social anxiety and somatic symptoms (43). previous study shows that somatic symptoms are common among adolescents and frequently prompt clinical visits (44). This may be related to parental concern and awareness. The study revealed that adolescents with social anxiety reported greater severity of somatic symptoms compared to controls (45) and that girls were at greater risk compared to boys (42). It’s worth noting that parents may not always discern their children’s mental health issues, especially mood disorders, and thus may not seek professional assistance. Conversely, adolescents tend to vocalize somatic complaints, which often prompts parental intervention. This suggests that adolescents may utilize somatic complaints as a means of expressing their emotional struggles and seeking support within the school environment (46). The various effects of unconscious impulses take the form of psychic and somatic symptoms and of impulsive behavior. Adolescents have poor emotional regulation and are prone to impulsive behaviors such as non-suicidal self-injury (47). Furthermore, school absenteeism is a perpetuating factor of functional somatic symptoms in adolescents (48).

The edge of “academic anxiety” and “social anxiety” also play an important role in this network. Students are constrained from participating in social, vocational, and recreational activities, which may impede their peer interactions (49). This limitation in social engagement can exacerbate social anxiety. Additionally, employing problem-solving-focused coping mechanisms, as opposed to emotion-focused strategies, may be instrumental in reducing social anxiety (50).

### 4.1 Limitation

When interpreting the results, the following limitations should be taken into account. Firstly, the study was cross-sectional and could not verify causality. Also, it could not determine the direction of the edges of the network. Secondly, this study was only conducted in one city and cannot represent the characteristics of the whole country. Thirdly, the network structure of our study is specific to the questionnaires we used. Thus, different questionnaires could produce a different network structure.

In addition, we chose only one time point (senior grade 3) and lacked longitudinal data. Future research should be carried out on a larger scale and further verified using different testing tools. The variability in the structure of the mental health symptom network by gender, ethnicity, and age was investigated in students at different stages of high school.

## 5 Conclusion

Despite the above limitations, our study still has some advantages. In this study, we investigated the network structure of MHT symptoms in senior grade three students and found that the core symptom of mental health is somatic symptom rather than the most prominent academic anxiety. These discoveries can advance the knowledge of mental health status in senior grade three students and provide some implications (i.e., targeting symptoms with the highest expected influence) for clinical prevention and intervention to address the mental health needs of this special group.

For students, they should find a suitable way to release pressure, maintain a good mental state, and rationally organize their studies and life, while schools and families should also pay attention to students’ mental health and provide necessary support and help.

## Data availability statement

The raw data supporting the conclusions of this article will be made available by the authors, without undue reservation.

## Ethics statement

The studies involving humans were approved by the Chinese Clinical Trial Ethics Committee. The studies were conducted in accordance with the local legislation and institutional requirements. Written informed consent for participation in this study was provided by the participants’ legal guardians/next of kin.

## Author contributions

GF: Formal analysis, Supervision, Writing – original draft, Writing – review & editing. YW: Methodology, Funding acquisition, Formal analysis, Writing – review & editing. HY: Methodology, Formal analysis, Writing – review & editing. NY: Writing – review & editing, Visualization, Data curation. SZ: Supervision, Writing – original draft, Writing – review & editing.
